# Prevalence of prurigo nodularis in the United States

**DOI:** 10.1016/j.jdin.2023.12.013

**Published:** 2024-03-22

**Authors:** Shawn G. Kwatra, Jorge Puelles, Oth Tran, Matthew Brouillette, Timothy Lillehaugen, Varsha Parthasarathy, Junwen Deng, Christophe Piketty, Sylvie Gabriel

**Affiliations:** a Johns Hopkins University School of Medicine, Baltimore, Maryland; b Galderma, Zug, Switzerland; c IBM Watson Health, Cambridge, Massachusetts; d Boston Scientific, Marlborough, Massachusetts; e Pfizer Inc., New York, New York; f Merative (formerly IBM Watson Health), Cambridge, Massachusetts; g Department of Dermatology, Johns Hopkins School of Medicine, Baltimore, Maryland

**Keywords:** epidemiology, incidence, itch, prevalence, prurigo nodularis, pruritus, skin diseases

*To the Editor:* Prurigo nodularis (PN) is a rare chronic inflammatory skin condition characterized by severe itch. Although the National Organization for Rare Diseases and Genetic and Rare Disease Information Center classifies PN as a rare disease,^1^ there is a paucity of data related to the prevalence of PN in the United States.^2^, ^3^, ^4^ Estimates from the few currently reported studies are highly variable, with prevalence rates for PN in adult patients ranging from 32.7 to 72 per 100,000 people.^2^, ^3^, ^4^ This study aims to confirm the classification of PN as a rare disease defined as fewer than 200,000 people affected nationwide by estimating the real-world prevalence of PN in the United States in 2019 and standardizing to the US population. This cross-sectional analysis used Merative MarketScan Commercial and Medicare claims databases which include patients with employer-sponsored private health insurance (Eczema area and severity index [EASI]). Patients were required to have ≥2 diagnoses for PN (the International Classification of Diseases, Tenth Revision, Clinical Modification L28.1) between 10/1/2015 and 12/31/2019 and continuous enrollment in the 12 months prior to 12/31/2019 (cross-sectional date). Period prevalence rates for patients with EASI were standardized to the US population by applying MarketScan National Weights. National weights allow researchers to project the MarketScan population to the US population with EASI. The person-level weights were created using the American Community Survey conducted by the US Census Bureau by estimating the number of people with EASI.^5^ Demographics were measured on the cross-sectional date and clinical characteristics were evaluated during baseline. Categorical variables were presented as the count and percentage of patients in each category, while continuous variables were summarized by providing the mean and standard deviation. A total of 6003 patients (82.6% Commercial and 17.4% Medicare) were selected for analysis (mean [SD] age 54[15.5], 54.8% female) (Fig 1). The observed period prevalence rate of PN in the MarketScan study population was calculated as 34 patients per 100,000 people. Observed period prevalence of PN was projected to the commercially insured, Medicare-insured, and combined EASI US populations using MarketScan person-level national weights. It was found that PN affects an estimate of 52.9 patients per 100,000 people with EASI. Specifically, it affects an estimate of 28.7 per 100,000 people in the commercially insured population and 127.1 per 100,000 people in the Medicare-insured population. By applying the prevalence rate of 52.9 per 100,000 people to the entire US population, 170,803 individuals were estimated to have PN in 2019. Results for prevalence of PN in the United States are presented in Table I. The prevalence estimates from the current analysis are in line with other estimates in US and non-US populations^2^, ^3^, ^4^ but utilized a more specific and robust definition of PN (requiring at least 2 diagnoses for PN that are at least 30 days apart and during a time period after which the specific diagnosis code for PN became available).^2^, ^3^, ^4^ This analysis confirms the designation of PN as a rare disease, as prevalence falls below the 200,000-person threshold established by the 1983 Orphan Drug Act,^1^ and provides real-world prevalence of PN in the United States.

**Fig 1:**
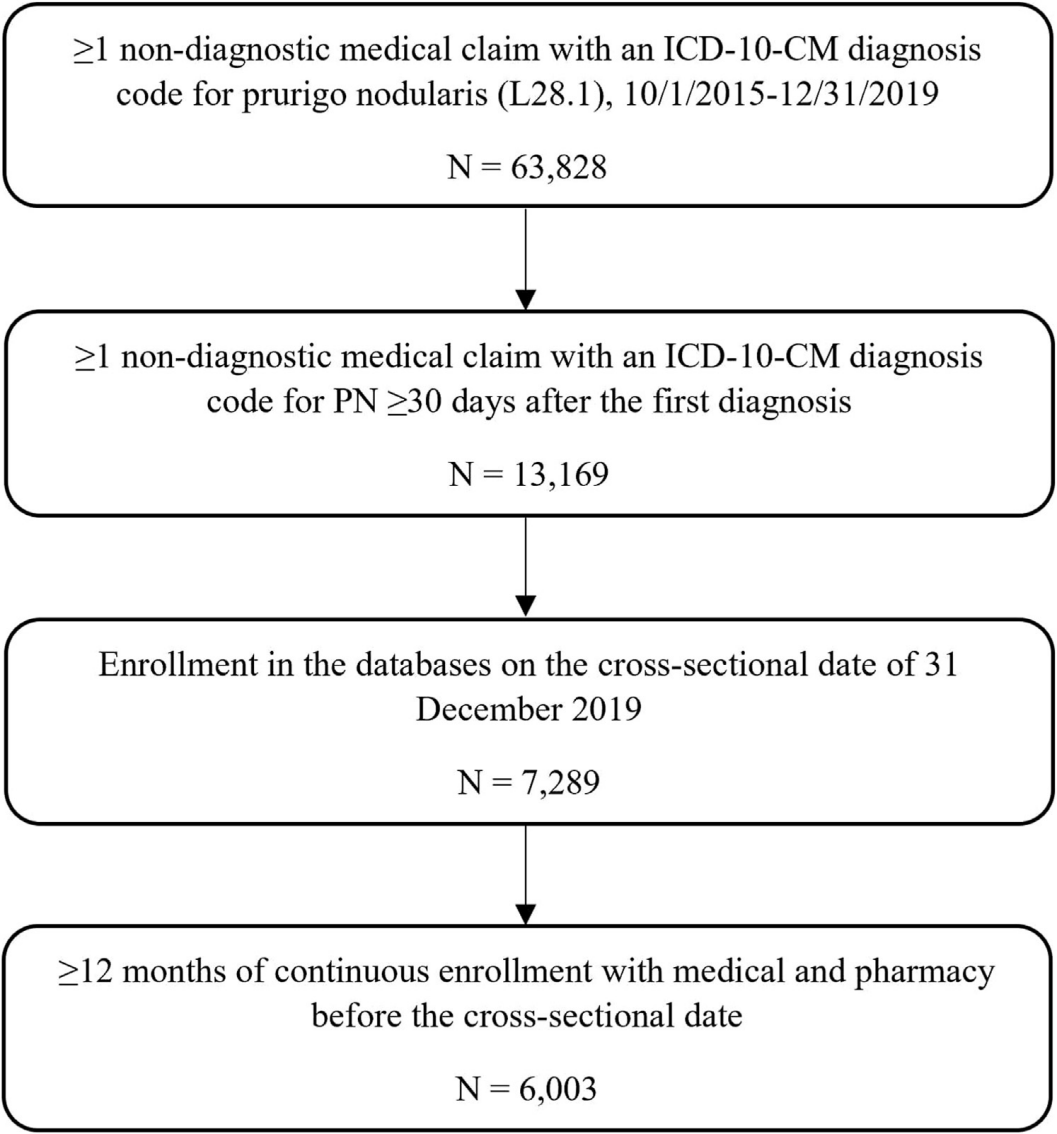
Prurigo nodularis patient selection. *PN*, Prurigo nodularis.

**Table I:** Estimated prevalence of prurigo nodularis in the United States

	Cross-sectional study sample (with continuous enrollment for the entire 2019)	Projected entire commercially insured population	Projected entire Medicare-insured population	Projected entire commercial and Medicare-insured population	Projected entire US population
Estimated total population, *N*	17,662,914	178,919,000	58,327,000	237,246,000	323,121,000
Estimated PN population, *N*	6003	51,273	74,137	125,409	170,803
Prevalence per 100,000 people, *N*	34.0	28.7	127.1	52.9	52.9

Period prevalence rate of PN in the study population was estimated by dividing the number of patients diagnosed with PN in the cross-sectional study population (numerator) divided by the number of individuals in the MarketScan databases with continuous enrollment for all 12 months of 2019 (denominator). Period prevalence rates were standardized to the US population in 2019 by applying MarketScan National Weights. National weights allow researchers to project the MarketScan population to the US population with employer-sponsored private health insurance.

*PN*, Prurigo nodularis.

## Conflicts of interest

Dr Kwatra received research grants from Galderma. Drs Puelles and Gabriel are employed by Galderma. Authors Tran and Brouillette were employed by IBM Watson Health at the time the study was conducted. Author Lillehaugen is employed by Merative. Drs Parthasarathy, Deng, and Piketty have no conflicts of interest to declare.
